# COVID-19 and Quarantine: Expanding Understanding of How to Stay Physically Active at Home

**DOI:** 10.3389/fpsyg.2020.566032

**Published:** 2020-10-29

**Authors:** Alberto Souza Sá Filho, Thiago Gottgtroy Miranda, Carolina Cavalcante de Paula, Silvio Roberto Barsanulfo, Diogo Teixeira, Diogo Monteiro, Luis Cid, Claudio Imperatori, Tetsuya Yamamoto, Eric Murillo-Rodriguez, Sandra Amatriain Fernández, Henning Budde, Sergio Machado

**Affiliations:** ^1^Post Graduate Program of University Center of Anápolis (UniEVANGÉLICA), Anápolis, Brazil; ^2^Physical Education Department of Universidade Paulista (UNIP–Campus Flamboyant), Goiânia, Brazil; ^3^Department of Cell and Developmental Biology, Institute of Biomedical Sciences (ICB), University of São Paulo (USP), São Paulo, Brazil; ^4^Faculty of Physical Education and Sport, Universidade Lusófona de Humanidades e Tecnologias (ULHT), Lisbon, Portugal; ^5^Intercontinental Neuroscience Research Group, Mérida, Mexico; ^6^Department of Human Kinetics, ESECS, Polytechnic of Leiria, Leiria, Portugal; ^7^Research Center in Sport, Health and Human Development (CIDESD), Vila Real, Portugal; ^8^Sport Science School of Rio Maior (ESDRM-IPSantarém), Rio Maior, Portugal; ^9^Cognitive and Clinical Psychology Laboratory, Department of Human Science, European University of Rome, Rome, Italy; ^10^Graduate School of Technology, Industrial and Social Sciences, Tokushima University, Tokushima, Japan; ^11^Escuela de Medicina, División Ciencias de la Salud, Universidad Anáhuac Mayab, Yucatán, Mexico; ^12^Faculty of Sport Sciences and Physical Education, University of A Coruña, A Coruña, Spain; ^13^Faculty of Human Sciences, Medical School Hamburg, Hamburg, Germany; ^14^Laboratory of Physical Activity Neuroscience, Physical Activity Sciences Postgraduate Program of Salgado de Oliveira University (PPGCAF/UNIVERSO), Niterói, Brazil; ^15^Laboratory of Physical Activity Neuroscience, Neurodiversity Institute, Queimados, Brazil

**Keywords:** COVID-19, HIIT (high intensity interval training), aerobic exercise, Tabata protocol, sedentarism

## Current Scenario

The coronavirus disease 2019 (COVID-19) is today the biggest public health challenge in the world (Park, 2020). The first case of COVID-19 was diagnosed on December 8, 2019, in Hubei province, China. From that day, in just over 3 months, the virus has spread to more than 177 countries/areas/territories around the world, with more than 266,073 confirmed cases and 11,184 deaths, according to WHO on March 21, 2020 (WHO, 2020). The most common clinical manifestations of COVID-19 are mild flu-like illness, potentially lethal acute respiratory distress syndrome, or fulminant pneumonia. As a result, numerous countries have decided to implement (some by government decrees, as well as martial laws) the establishment of mandatory social distance in a family environment, closing non-essential commercial environments, in an attempt to reduce the peak of the infection curve (Lewnard and Lo, 2020).

We know that a large part of the world population is far from the minimum conditions of physical exercise recommended by the American College of Sports Medicine (ACSM) to improve the health component (Katzmarzyk et al., 2019). This fact would give important relevance to the level of physical activity exercised by the population throughout the day. However, once the extreme hypokinetic behavior is implemented as a result of the quarantine, a cycle of perverse events begins, making part of the population more vulnerable to the deleterious effects of acute and chronic diseases, including respiratory tract infections (Hall et al., 2020).

In a recent position paper presented by Chen et al. (2020), the authors try to propose to the general population to continue exercising regardless of the current moment the world is living. In fact, as the authors mention, based on other researchers, “anything is better than nothing,” and the sedentary lifestyle is something that should not be encouraged, i.e., any energy expenditure added to the routine of these people would be significant. Recommendations for the population to keep regularly active highlight only a minimum applicable technical basis, without presenting any suitable parameters for carrying them out. From the initial positioning of Chen et al. (2020), the ACSM *via* publication on the website of the journal *Medicine* & *Science in Sports* & *Exercise* (ACSM, 2020; WHO, 2020), as well as other institutions (ACSM, 2020; WHO, 2020), expanded the proposal about the practice of physical exercise to be performed at home. Tasks such as brisk walking, up and down stairs, dance, jump rope, yoga exercises, and bodyweight strength training are also recommended for indoor workout (Table 1).

**Table 1:** Recommendations for exercising at home.

	**Aerobic exercises**	**Strength exercises**
Conception	Prolonged or short term exercises using large muscle groups	Localized exercise with body weight, or free weight Exercises (include all major muscle)
Configuration	Merging one or more strength component with an aerobic component (see Table 2)	
Frequency	3–5 days/week (consecutive days for high levels of fitness)	
Time	10–30 min a day. This can be accumulated continuously or in shorter 10-min blocks	
Intensity	Moderate effort (40–59% of heart rate—HR) for long workouts (RPE 3–4) or for lower levels of conditioning; Moderate to high effort (60–85% of HR)—for intermediate workout times (RPE 4–6); High effort (>85% of HR)—for short workouts (RPE >7)	
Volume	150 or more min/week are required. 4–6 sets of 6–20 repetitions for selected exercise. 3–5 exercises for workout	
Workout form	(a) Mobility and warm up (5–10 min); (b) core or strength (5–10 min); (c) multimodal workout (5–20 min)	

## Expanding Recommendations for Practicing Exercise at Home

The suggestions proposed by the ACSM (2020), WHO (2020), although more consistent, still do not offer a concrete logic to be applied and controlled. Moreover, the statement “Some activity is better than none” makes more sense when we talk about people practicing any physical activity at a beginner level, therefore merely minimally physically active (Chen et al., 2020). Thus, for practitioners of physical exercise at levels that require moderate to high metabolic and strength demand, or even athletes, these would be susceptible to detraining. According to the basic premises related to training, an ideal stimulus must be administered for the adaptation to occur properly, and that condition may not be prioritized with such positions.

Considering the theoretical rationale prevalent in the literature, we believe that the suggestions proposed can be expanded in order to integrate groups of individuals who have moderate to high physical conditioning and not only sedentary individuals, providing better guidance on how to proceed during the quarantine period and offering the possibility of follow-up training even after the quarantine has ended. For such, the conviction that traditional strategies of aerobic endurance exercise may not be a suitable strategy for application in the residential environment (understanding that majority of the population does not have stationary bikes, arm or rowing ergometers at home), so the interval exercise pattern should be primarily stimulated, with or without the use of any viable resource of overload implementation. In combination with this proposal, the concomitant increase in intensity (vigorous to high intensity) is also essential to promote adaptive results independently of the initial fitness level.

The effects of high-intensity interval training (HIIT) are well-established in the literature for healthy people (Gormley et al., 2008) and those with some comorbidity (obesity, heart disease, diabetics) (Ballesta Garcia et al., 2019; Taylor et al., 2019). More recently, the effects of HIIT have been presented in sedentary individuals, suggesting that the application of interval exercises would be viable, consolidating itself as an important strategy for health promotion (Dorneles et al., 2019; Reljic et al., 2019). The literature shows significant physiological responses derived from different types of interval protocols (Paoli et al., 2012; Buckley et al., 2015; Box et al., 2019), and the improvement in performance seems to be related to the physiological mechanism of inducing mitochondrial biogenesis from the expression of the PGC-1 alpha transcription co-activator, as well as catalyzing enzymes of both the glycolytic and oxidative systems (Gibala et al., 2006; Gibala, 2009). Such adaptations promote greater efficiency metabolic rate in energy production and buffering capacity.

In a new perspective, evidence indicates that HIIT performed with body weight can promote significant adjustments in strength, hypertrophy (Kikuchi and Nakazato, 2017), and the cardiorespiratory system. For instance, the Tabata protocol would fit as an interesting tool to be performed at home (Tabata et al., 1996). Basically, it consists of performing stimulus 8 × 20 s interspersed with 10-s recovery, a total of 4 min. The protocol is still performed more than once during an exercise session and with different exercise compositions. Emberts et al. (2013) reported mean values of 74% of VO_2max_ [rate perceived exertion (RPE) averaged 15.4 ± 1.3] and 86% of HR_max_ (156 ± 13 bpm) during two types of Tabata workouts (e.g., mountain climbers, push-ups, split squat, box jumps, burpees, squats, lunges, Russian twist). This level of workout is a sufficient stimulus to generate adaptations to the cardiorespiratory component, and these data are superior to the recommendations proposed by the ACSM. Moreover, the increase in intensity seems to be the key to maintain the gains obtained before COVID-19 (Hickson et al., 1985).

To better target the perspective postulated here in our article, as well as to better interpret the designs positioned in Table 2, Buckley et al. (2015) proposed a high-intensity multimodal training format as a way to reduce the time required for multiple adaptations. For this, the authors compared the physiological responses of the traditional HIIT performed in a rowing ergometer versus multimodal training, involving analysis of different manifestations of strength, in addition to maximum aerobic power and anaerobic capacity. Thirty-two recreational trained participants performed 60 s “all out” and a 3-min recovery (total of 4 min per series). The multimodal HIIT protocol was configured as follows: a strength exercise for 4–6 repetitions, an accessory movement for 8–10 repetitions, and a metabolic component conducted all out for the remainder of the 60 s. The results were significantly promising, resulting in similar responses in aerobic and anaerobic performance tests; however, multimodal HIIT showed significant improvement in all parameters of different manifestations of strength.

**Table 2:** Proposal of exercises for workouts configuration.

**Upper body**	**Lower body**
Push up (or adapted)	Hip trust (on the ground)
Pull ups (or adapted)	Squat or split squat
Handstand push up (or inverse press on chair)	Sumo squat
Ball throw (or adapted)	Lunge (or walking lunge)
Dips on chair or box	Pistol (advanced)
Shoulder push up (on the ground)	Good morning
Adapted bent over row (pulling a towel)	Adapted deadlift or single leg deadlift
**Aerobic demand**	**Core**
Jumping jacks	Hollow body (or hold)
Jump rope (single or double under)	Arch body (or hold)
Burpees	Sit ups
Box jump (stairs jump)	Plank or side plank
Box jump over (on chair)	Turkish get up
Skipping (performed in a hallway)	Russian twist
Sprawl	Mountain climber

It is suggested, therefore, that the configuration of multimodal workouts be constructed in a similar way to that reported in the literature, and the control of exercise overload (internal load) would be performed based on the RPE (0–10 in combination with session time (Foster et al., 2001). Table 2 shows a coherent exercise division format, and Table 3 shows examples of training session configurations.

**Table 3:** Examples of training session configurations based on intensity control from internal load.

**Lower body program**			**Upper body program**		
**Warm up**	**(5 min)**	**RPE 3–4**	**Warm up**	**(5 min)**	**RPE 3–4**
2 sets	Specific mobility (hip)		2 sets	Specific mobility (shoulders)	
	30×	Jumping jacks		50×	Single under
	50×	Jump rope		50×	Skipping
Core	(10 min)	RPE 4–6	Strength	(10 min)	RPE > 7
3 sets	15×	Hollow body	3 sets	8–10×	Adapted handstand push up
	30 s	Arch hold		8–10×	Pull up
Workout	(15 min)	RPE 4–6	Workout	(15 min)	RPE > 7
AMRAP	20×	Walking lunges	MAX RFT	20×	Push up
	20×	Sprawl		20×	Dip on box
	20×	Split squat		20×	Mountain climbers
	50×	Double under		20×	Burpees

## Can Exercise Intensity Compromise the Immune System?

Finally, establishing the relationship between the stresses generated from physical exercise at home and the immune system is an important point to be considered during this quarantine period (Amatriain-Fernandez et al., 2020a,b). Nieman (2007) proposes an open window of alteration of the immune system after physical exercise, and such manifestation would occur with significant magnitude in the face of long-lasting endurance, such as in a marathon, or also in the face of extremely heavy efforts. However, little is known about the immune responses to short interval exercise, but current evidence suggests that HIIT seems to be beneficial for the immune system (Bartlett et al., 2017, 2018; Born et al., 2017; Durrer et al., 2017; Dorneles et al., 2019; Steckling et al., 2019; Khammassi et al., 2020), although evidence still points to a higher increase in the percentage of leukocytosis after HIIT exercise (Jamurtas et al., 2018).

So, Bartlett et al. (2017) investigated in 27 sedentary adult individuals the potential of immune response induced by continuous aerobic training of moderate intensity (MICT) and HIIT (volume 57% smaller). After 10 weeks, there was a significant improvement in the capacity of bacterial phagocytosis by neutrophils (+16 vs. +15%, respectively, for HIIT and MICT) and monocytes (14 vs. 19%, respectively, for HIIT and MICT) for both training groups. Also with a more recent perspective, Born et al. (2017) demonstrated that HIIT, in addition to the superior adaptive responses on the ability to perform exercise (time to exhaustion—*p* = 0.02; VO_2Max_–*p* = 0.01), induced functional immunoglobin-A adaptations following 4 days of training in recreational adult runners. Furthermore, HIIT promotes similar inflammatory responses after exercise compared to traditional endurance training, suggesting its viability as a training strategy (Kaspar et al., 2016; Bartlett et al., 2017). However, an adequate progression of intensity is suggested to avoid deleterious effects due to high doses of exercise. In the workout model recommended here, despite the fact that it is called high-intensity interval exercise, the effective physiological impact (product of volume vs. intensity) is reasonably small (main workout). Moreover, such proposals mainly focus on recreational trained people. In line with this, several studies have shown significant findings in favor of HIIT protocols when compared to moderate-intensity exercise, showing how the immunological system responds to vigorous to high-intensity training with very short duration (Table 4).

**Table 4:** Main positive and negative results from the perspective of HIIT and the changes resulting from this training model.

Durrer et al. (2017)	
Objective:	To determine the impact of a single session of HIIT on cellular, molecular, and circulating markers of inflammation in individuals with Type 2 Diabetes (T2D)
Participants:	Participants with T2D (*n* = 10) and healthy (HC) age-matched controls (HC; *n* = 9)
Intervention:	Acute bout of HIIT (7× 1-min at 85% maximal aerobic power output), separated by 1-min recovery on a cycle ergometer
Measures:	Blood samples Pre, Post, and 1-h Post. Inflammatory markers on leukocytes and tumor necrosis factor (TNF)-α
Outcome:	(a) significantly ↓ levels of toll-like receptor (TLR); expression on both classical and CD16^+^ monocytes assessed at Post and 1-h Post compared with Pre; (b) significantly ↓ LPS-stimulated TNF-α release in cultures at 1-h Post; (c) significantly lower levels of plasma TNF-α at 1-h Post. There were no differences between T2D and HC except for a larger decrease in plasma TNF-α in HC vs. T2D
Bartlett et al. (2018)	
Objective:	Determine whether 10 weeks of a walking-based HIIT program would be associated with health improvements. Assess whether HIIT was associated with improved immune function, specifically antimicrobial/bacterial functions of neutrophils and monocytes
Participants:	Twelve physically inactive adults
Intervention:	3 × 30-min sessions/week of 10 ≥ 60-s intervals of high intensity (80–90% VO_2reserve_), and rest of 50–60% VO_2reserve_
Measures:	Pre- and post-aerobic and physical function; self-perceived health; C-reactive protein (CRP), and erythrocyte sedimentation rate (ESR); plasma interleukin (IL)-1β, IL-6, chemokine (C-X-C motif) ligand (CXCL)-8, IL-10, and tumor necrosis factor (TNF)-α concentrations; and neutrophil and monocyte phenotypes and functions
Outcome:	VO_2max_ ↑9%; Neutrophil migration toward CXCL-8, phagocytosis of Escherichia coli, and ROS production all increased following training. The frequency of differentiation 14-positive (CD14^+^)/CD16^+^ monocytes was reduced, with both non-classical (CD14^dim^/CD16^bright^) and intermediate (CD14^bright^/CD16^positive^) monocytes being reduced; Expression of Toll-like receptor 2 (TLR2), TLR4, and HLA-DR was reduced and monocyte phagocytosis of E. coli increased
Bartlett et al. (2017)	
Objective:	Compared the impact of HIIT and moderate-intensity continuous training (MICT) on immune function in sedentary adults
Participants:	Twenty-seven healthy sedentary adults
Intervention:	HIIT (>90% maximum heart rate) or MICT (70% maximum heart rate) group training program
Measures:	VO_2peak_, neutrophil and monocyte bacterial phagocytosis and oxidative burst, cell surface receptor expression, and systemic inflammation were measured before and after the training
Outcome:	Total exercise time was 57% less for HIIT; Significantly improved VO_2peak_ for both; Oxidative burst and monocyte phagocytosis and percentage of monocytes producing an oxidative burst were ↑ by training similarly; Expression of monocyte but not neutrophil CD16, TLR2, and TLR4 was ↓ by training similarly in both groups; No differences in systemic inflammation were observed for training
Khammassi et al. (2020)	
Objective:	Compare the effects of HIIT and moderate-intensity continuous training (MCT) on hematological biomarkers in active young men (9 weeks/3 training per week)
Participants:	Sixteen men aged 18–20 years were randomly assigned to HIIT or MCT group
Intervention:	HIIT: (30 s at 100% of maximum aerobic velocity/30 s rest at 50%); MCT sessions were matched for workload based on the total distance in HIIT
Measures:	VO_2max_; red blood cell, hemoglobin, hematocrit, mean corpuscular volume, mean corpuscular hemoglobin, mean corpuscular hemoglobin concentration, leukocyte, neutrophil, lymphocyte, monocyte, and eosinophil count
Outcome:	No significant change was observed in maximal aerobic velocity and estimated VO_2max_ in both groups; Leukocyte, lymphocyte, neutrophil, and monocyte count showed significant improvements in response to the MCT; The MCT intervention favored an increase in the number of immune cells
Jamurtas et al. (2018)	
Objective:	Evaluated the effects of HIIT on hematological profile and redox status compared with those following traditional continuous aerobic exercise (CET)
Participants:	Twelve healthy young men participated in a randomized crossover design under HIIT and CET
Intervention:	HIIT: 4x 30-s sprints on a cycle-ergometer/4 min of recovery. CET: 30-min cycling on a cycle ergometer at 70% of their VO_2max_
Measures:	Blood was measured at baseline, immediately after, 24, 48, and 72 h post-exercise and was analyzed for complete blood count and redox status (thiobarbituric acid reactive substances, [TBARS]; protein carbonyls, [PC]; antioxidant capacity total, [TAC]; catalase and uric acid)
Outcome:	White cells ↑ immediately post-exercise (HIIT: 50% and CET: 31%, respectively); HIIT ↑+22% PC post-exercise compared to CET; HIIT ↑+16% TAC immediately post-exercise and at 24 h post-exercise (11%), while CET ↑ TAC only post-exercise (12%, *p* < 0.05); Both HIIT and CET ↑ uric acid immediately post- (21 and 5%, respectively) and 24 h (27 and 5%, respectively); There were no significant changes for TBARS and catalase following either exercise protocol
Born et al. (2017)	
Objective:	Evaluate the mucosal immune function and circadian variation of salivary cortisol, Immunoglobin-A (sIgA) secretion rate and mood during a period of high-intensity interval training (HIIT) compared to long-slow distance training (LSD)
Participants:	28 Recreational male runners
Intervention:	9 sessions (3 weeks); HIIT: 4 × 4 min of running at 90–95% of max HR/3 min rest; LSD: continuous running at 70–75% of max HR for 60–80 min
Measures:	Salivary cortisol and immunoglobin-A (sIgA); VO_2Peak_ and Performance
Outcome:	HIIT = longer time-to-exhaustion and ↑VO_2peak_ compared to LSD, sIgA secretion rate was higher on the last day of training, as well as the area under the curve (AUCG) higher on the first and last day of training and follow-up compared to the LSD. The AUCG for cortisol remained unaffected on the first and last day of training but increased on the follow-up day with both, HIIT and LSD. sIgA secretion rate with the HIIT indicates no compromised mucosal immune function
Bartlett et al. (2020)	
Objective:	Determine if neutrophil functions could be improved in association with changes in fitness and metabolic parameters in older adults at risk for Type 2 Diabetes Mellitus using 10-weeks of low volume high-intensity interval exercise training (HIIT)
Participants:	Ten older sedentary adults with prediabetes completed 10 weeks of a supervised HIIT program
Intervention:	10x 60 s intervals at 80–90% Heart rate reserve/50–60% HRR rest
Measures:	Before and after training, VO_2peak_, glucose and insulin sensitivity, neutrophil chemotaxis, bacterial phagocytosis, reactive oxygen species (ROS) production, and mitochondrial functions were assessed (VO_2peak_ and neutrophil functions were compared to six young (23 ± 1 years) healthy adults)
Outcome:	Significant ↓ in fasting glucose and insulin were accompanied by ↑ glucose control and insulin sensitivity; VO_2Peak_ ↑ 16 ± 11%; Following training, chemotaxis phagocytosis and stimulated ROS ↑ while basal ROS ↓ similar to levels observed in the young controls; mitochondrial functions ↑ toward those observed in young controls, ↓ the deficit of the young controls between
Dorneles et al. (2019)	
Objective:	To verify the effect of 1 week of high-intensity interval training (HIIT) on the peripheral frequency of T helper subsets and monocyte subtypes
Participants:	Seven sedentary obese men
Intervention:	One week of HIIT (3 ×/week)−10 bouts of 60 s (85–90%HR_max_) alternated with 75 s of recovery (50%HR_max_)
Measures:	Blood samples before and 24 h after the last session for phenotypic analysis of T cells and monocytes
Outcome:	After 1 week of HIIT, an ↑ in VO_2Peak_. Short-term HIIT ↑ Treg (CD4^+^ CD25^high^ CD127^low^); and mTreg cells (CD4^+^ CD25^+^ CD39^+^); No statistical difference was observed in other immune cell phenotypes analyzed
Steckling et al. (2019)	
Objective:	Effects of HIIT on systemic levels of inflammatory and hormonal markers in postmenopausal women with metabolic syndrome (MS)
Participants:	Fifteen postmenopausal women with MS
Intervention:	Treadmill running 3× per week, for 12 weeks. 4× 4 min intervals at 90% HR_max_, with 3 min active recovery at 70% HR_max_
Measures:	Body composition, VO_2max_, serum plasma levels of cytokines (levels of IL-1b, IL-6, IL10, IL-18, TNF-a, interferon-gamma—IFN-c), nitrate and nitrite (NOx) levels, and adiponectin, resistin, leptin, and ghrelin were determined along the intervention
Outcome:	VO_2max_ and anthropometric parameters were ↑ after HIIT, while ↓ levels of proinflammatory markers and ↑ levels of interleukin-10 (IL-10) were also found. Adipokines were also modulated after 12 weeks or training. The mRNA expression of the studied genes was unchanged after HIIT
Kaspar et al. (2016)	
Objective:	To compare effect of single-bout endurance (ET) and HIIT on the plasma levels of 4 inflammatory cytokines and C-reactive protein and insulin-like growth factor
Participants:	Seven healthy untrained volunteers
Intervention:	HIIT: 6 sets of 30 s of all-out supramaximal intensity cycling; ET: 45 min of ergometer cycling at a moderate intensity, which was calculated at 62.5% of Max HR
Measures:	Plasma samples for the interleukins (IL), IL-1β, IL-6, and IL-10, monocyte protein-1 (MCP-1), insulin growth factor 1 (IGF-1), and C-reactive protein (CRP)
Outcome:	ET: significant acute and long-term inflammatory response with ↓ decrease at 30 min after exercise in the IL-6/IL-10 ratio (−20%) and a ↓ of MCP-1 (−17.9%); There were no significant changes in the plasma levels of CRP, IL-1, and IGF-1 from baseline to either 30 min or 2 days after the intervention

## Future Perspectives

It is reasonable to think that HIIT can also be adjusted to improve physical fitness and health in individuals with low levels of fitness (Gormley et al., 2008), as well as for overweight and obese people, according to the trend facing this pandemic (Wewege et al., 2017). First, it is important to understand that the term high intensity should not necessarily be interpreted as a high effort (that would generate limiting condition), since the effort depends on the ratio between intensity and time. In the case of protocols with neuromuscular characteristics, it is possible to establish a suitable threshold for each fitness pattern, mainly controlling the pace with which the movements are performed or the time spent in each stimulus. For cyclic aerobic exercise, the external load, related to the percentage level of VO_2Max_ required by the coach, is in high physical demand, while the internal load, referring to internal perceptions and changes, can modulate a perceived effort to tolerable levels (Foster et al., 2001). Thus, considering the non-prolonged exposure to high-intensity stimuli, we were able to produce significant results for the cardiorespiratory component (Buchheit and Laursen, 2013), as well as important functional adaptations to the immune system (Bartlett et al., 2017), and promote greater adherence to exercise by individuals with a lower level of fitness (Hartman et al., 2019). Therefore, HIIT is expected to be recognized from a safe and effective dose–response perspective (Taylor et al., 2019) as a potential tool for the improvement of the immune system and consequently for the prevention of respiratory diseases.

## Author Contributions

AS participated in the conception of the idea and complete writing of the article, along with SB, CdP, and TM. SM, DT, DM, LC, and CI participated in numerous reviews of this study. CI, TY, and SA participated in the suggestions and the final writing of the article and the adequacy and submission of the study. HB, EM-R, and SM were the main advisers and tutors of all trajectory of studies and designing all phases of the study. All authors contributed to the article and approved the submitted version.

## Conflict of Interest

The authors declare that the research was conducted in the absence of any commercial or financial relationships that could be construed as a potential conflict of interest.
